# Comparison of low load blood flow restriction and high load resistance training of the finger flexors in advanced level climbers: a pilot study

**DOI:** 10.3389/fphys.2026.1807492

**Published:** 2026-04-24

**Authors:** Vidar Andersen, Espen Hermans, Kristoffer Gunther Hansen, Tom Erik Jorung Solstad, Atle Hole Saeterbakken, Jiří Baláš, Gøran Paulsen, Nicolay Stien

**Affiliations:** 1 Faculty of Education, Arts and Sports, Western Norway University of Applied Sciences, Sogndal, Norway; 2 Faculty of Physical Education and Sport, Charles University, Prague, Czechia; 3 Norwegian School of Sport Sciences, Oslo, Norway; 4 Norwegian Olympic and Paralympic Committee and Confederation of Sports, Oslo, Norway

**Keywords:** climbing, fingerboard, occlusion, performance, strength training

## Abstract

**Introduction:**

The aim of the present pilot study was to compare the effects of low load blood flow restriction (LL-BFR) with high load resistance training (HL) in advanced level climbers.

**Methods:**

Twenty-two climbers were randomly allocated to LL-BFR or HL performing training twice per week for five weeks. Before and after the intervention the participants were tested in isometric pull-up (peak- and average force), maximal voluntary contraction (MVC) in a finger flexor exercise, finger endurance, forearm circumference, and climbing performance.

**Results:**

There were no group differences in any of the tests (p=0.346-0.891), however, both groups increased their average force in the pull-up (LL-BFR; 52 N, p=0.012, HL; 56 N, p=0.024), MVC (LL-BFR; 15 kg, p=0.008, HL; 17 kg, p=0.002), forearm circumference (LL-BFR; 0.8 cm, p=0.012, HL; 0.6, p=0.038) and climbing performance (LL-BFR; 13.5 moves, p=0.012, HL; 10 moves, p=0.003). No pre-post differences were observed for the peak force in the pull-up (p=0.132-0.376) or the endurance test (p=0.752-1.000).

**Discussion:**

In conclusion, resistance training of the finger flexors with HL or LL-BFR resulted in no between-group differences, with both interventions improving maximal strength, hypertrophy, and climbing performance, but not endurance.

## Introduction

Performance in climbing is influenced by several factors such as technique, tactics, psychology and physicality (Sanchez et al., 2019; Vasile et al., 2023; Medernach et al., 2024). Among the physical factors, finger strength is regarded as one of the most important enabling force production against small holds (Saul et al., 2019; Diez-Fernández et al., 2023). Consequently, specific training of the finger flexors to improve finger flexor strength has become a key focus in sport climbing training programs (Langer et al., 2023; Stien et al., 2023). Notably, the fingers are the most prevalent site of injury in climbing (Jones and Johnson, 2016; Grønhaug, 2018) and overuse seems to be one of the main causes (Barrile et al., 2022). Therefore, and importantly, increasing the training volume by initiating resistance training of the finger flexors should be done with caution as it also might lead to injuries.

Blood flow restricted (BFR) training has been suggested as an alternative to traditional resistance training (Patterson et al., 2019). BFR involves using a cuff to induce external pressure to a limb proximal to the working muscles. Thereby, arterial blood flow is restricted, while venous return is blocked (Scott et al., 2015). This technique creates a state of localized hypoxia and metabolic stress that accelerates neuromuscular fatigue development and prevents inter-set recovery, which triggers high or maximal motor unit recruitment even at low loads (Wernbom et al., 2008; Scott et al., 2016; Wernbom and Aagaard, 2020; Pignanelli et al., 2021; Simpson et al., 2025). This will eventually stimulate adaptations such as muscle hypertrophy and strength, as well as oxidative capacity (Loenneke et al., 2012; Lixandrão et al., 2018). The lower absolute loads reduce the mechanical strain on the affected joints, which in turn may reduce injury risks. Consequently, BFR training of the finger flexors could be a viable alternative to high load resistance training for climbers aiming to increase their finger flexor physical capacity and thereby their climbing performance.

To the best of our knowledge, only two previous studies have examined the chronic effects of BFR training in climbers (Held et al., 2023; Javorský et al., 2023). Held et al. (Held et al., 2023) reported favorable increases in grip- and arm endurance, but not strength, when conducting low intensity climbing with or without BFR in advanced climbers. Javorsky et al. compared five weeks of BFR resistance training focused on the finger flexors (30% of MVC) with high intensity (60% of MVC) without BFR in lower grade to intermediate climbers (Javorský et al., 2023). The authors reported no difference between the two training groups for any of the parameters, but increased force impulse in the 4-min all-out test for the group training with high intensity and no BFR. Of note, the study used a rather low training volume as the aim was to maintain, and not increase, climbing-specific strength and endurance. This may be of relevance for athletes, as low-load BFR training during tapering allows reduced training loads without compromising performance (Smith et al., 2025). Furthermore, Javorsky et al. (Javorský et al., 2023) did not directly measure climbing performance and the population consisted of lower grade to intermediate climbers, who more likely could benefit more from developing technical and mental skills rather than isolated finger flexor capacity. Therefore, there is a research gap in comparing the effects of low load BFR (LL-BFR) and high load (HL) finger flexor training on physical attributes and climbing performance in advanced climbers. Of relevance, there has been one cross-sectional study comparing the affective responses of specific finger flexor training with and without BFR in advanced level climbers (Andersen et al., 2023). Interestingly, the climbers reported the BFR- and high load resistance training to be similarly exertive, however, BFR was perceived as more discomforting and less enjoyable. Consequently, it is of great scientific and practical interest to examine and compare the longitudinal effects of BFR- and high load resistance training of the finger flexors, but also observe the feasibility of these training modalities over time. Therefore, the aim of the present study was to compare the effects of BFR- with high load resistance training of the finger flexors for maximal finger strength, finger endurance, hypertrophy and climbing performance in advanced level climbers.

## Methods and materials

### Study design

An experimental study design with within- and between- groups comparisons was used to investigate the chronic effects of finger flexor resistance training with and without blood flow restriction. All participants were randomized into finger flexor resistance training with either low load and BFR (LL-BFR) or high load without BFR (HL). Both groups conducted two training sessions per week over a period of five weeks. Furthermore, the participants were asked to continue their normal climbing training routines. All participants were tested pre and post in an isometric pull-up measuring peak (Fpeak) and average force (Favg), a maximal voluntary isometric contraction (MVC) on the fingerboard used in the intervention and an intermittent endurance (IE) test with a work: rest ratio of 7:3 seconds on the same fingerboard. Hypertrophy was measured as circumference of the dominant forearm and climbing performance measured as the number of moves in a pre-determined lead climbing route.

### Participants

The sample size was based on convenience as we tried to invite all climbers that fit the inclusion criteria in the nearby area to participate in the study. The recruitment process involved putting up flyers, holding information meetings for the local climbing club, reaching out to known possible participants, and putting up stands in the local climbing facilities to inform and recruit participants. To be included as a participant, one had to be over eighteen years old, free of injuries that could affect testing or training for the last six month, have a self-reported climbing ability within the advanced level (female: 15-20, male: 18-23) according to the International Rock Climbing Research Association (IRCRA) scale (Draper et al., 2015). In the end, twenty-two advanced-level climbers (twenty males and two females) volunteered and met the inclusion criteria for the study. Three participants withdrew as they perceived the BFR training too discomforting. Hence, nineteen climbers completed the intervention program and were included in the analysis (see **Table 1**). The study was assessed by the Regional Committee for Medical and Research Health Ethics, South-East Norway (2018/1345) and deemed to be outside the national criteria for a required ethical approval. The study was conducted according to the University College’s ethical guidelines, and the procedures for collecting and saving personal data were approved by the National Centre for Research Data (reference nr: 525536). All participants were informed orally and in writing about the protocol and procedures and had to sign an informed consent to be enrolled in the study.

**Table 1:** Anthropometrics, climbing level and training routines at baseline (mean ± standard deviation).

Descriptives	LL-BFR (n=8)	HL (n=11)
Age (years)	28.8 ± 3.8	25.4 ± 7.0
Height (meters)	1.81 ± 0.06	1.76 ± 0.12
Body mass (kilogram)	73.7 ± 7.3	66.6 ± 11.1
BMI (kilogram/meter^2^)	22.5 ± 1.8	21.3 ± 1.2
IRCRA grade	19.0 ± 3.3	18.3 ± 1.9
Bouldering (hours/week)	2.8 ± 1.0	2.7 ± 1.6
Lead climbing (hours/week)	2.4 ± 1.7	3.5 ± 5.7
Strength training (hours/week)	1.5 ± 1.5	1.0 ± 0.8
Endurance training (hours/week)	2.0 ± 1.9	3.6 ± 2.9

LL-BFR, low load blood flow restriction; HL, high load training.

### Testing procedures

Before all testing sessions, the participants were instructed to refrain from alcohol, climbing, and climbing specific strength training the last 48 hours in advance. The tests were conducted in the same order as they are described below, and by the same test leader in every test and training session.

### Anthropometrics and forearm circumference (Hypertrophy)

Before starting the warm-up, the participants reported their weekly training routines, experience, and climbing level. Thereafter, stature and body mass were measured in addition to the circumference of the forearm of the dominant hand. This measure was taken 30% proximal of the distance between the styloid process of the radius and the insertion of the biceps brachii muscle into the radial tuberosity (Meza-Valderrama et al., 2022). The measurement was taken while the participants sat relaxed with the arm hanging down. The measuring tape was placed close to the skin, without deforming or pressing on of the tissue. The measure was reported to the nearest millimeter. A previous study have shown acceptable reliability (CV = 6.8) when measuring arm circumference in advanced climbers (Ozimek et al., 2017).

#### Isometric pull-up tests

Before initiating the physical tests, all participants conducted a 15-minute warm-up consisting of progressive bouldering and sub-maximal hangs on a fingerboard. The participants were instructed to start out with easy bouldering on good holds for ten minutes, and to gradually increase the intensity to moderate for the last five minutes without getting fatigued.

**Figure 1:**
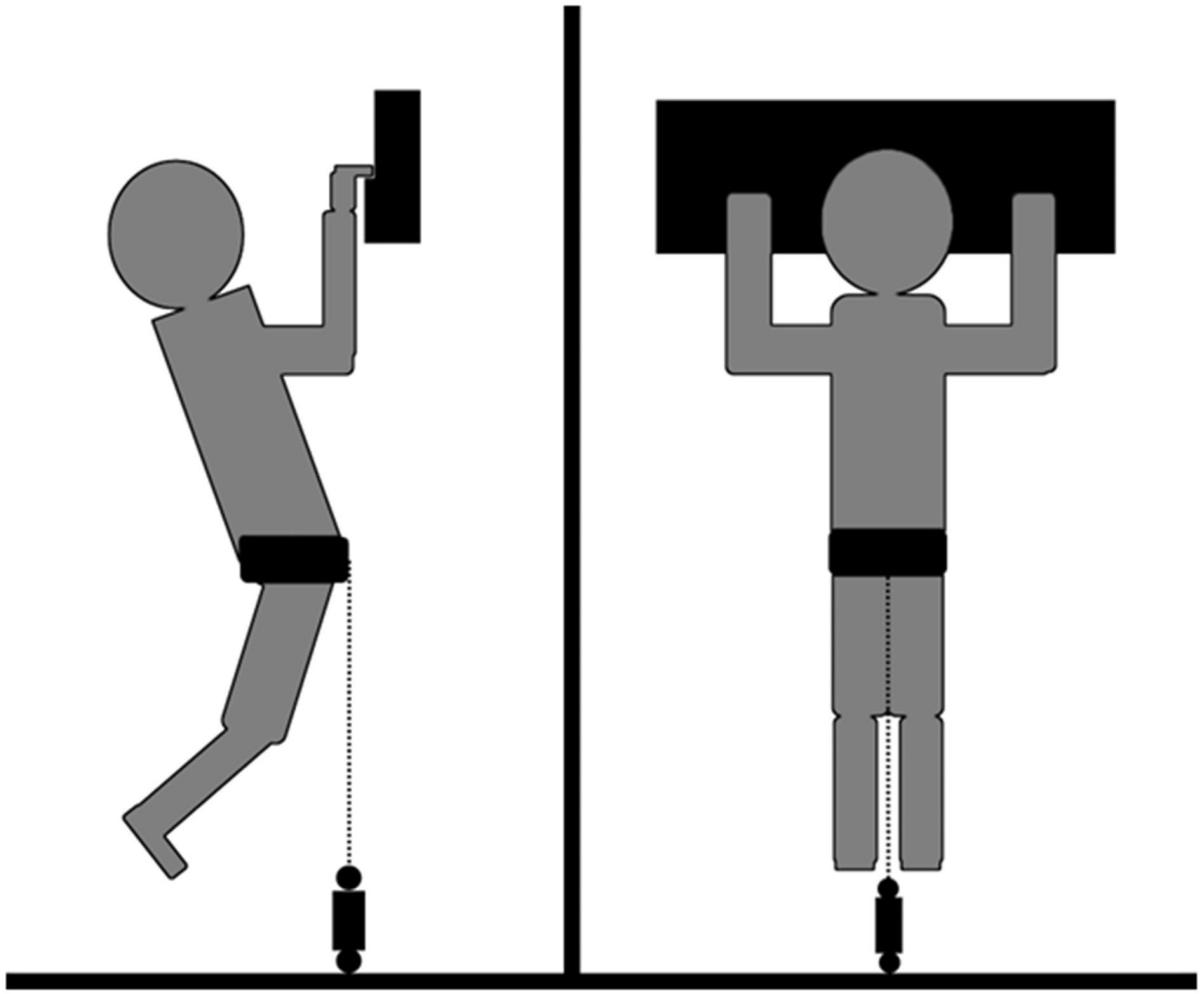
Schematic overview of the isometric pull-up test.

The isometric pull-up test (see **Figure 1**) was conducted in accordance with several earlier studies (Stien et al., 2021b; Stien et al., 2021a; Vereide et al., 2022). The test was conducted bilaterally hanging vertical on a 23mm rung. Force output was measured using a force sensor at 200Hz (Ergotest-Innovation-AS, 2015) through a static system consisting of an expansion bolt in the floor, the force cell, a daisy chain (Singing rock safety chain, 16mm) and a climbing harness (Arc’teryx, R320a climbing harness). The daisy chain was used for adjusting the length between the force sensor and the harness to achieve a 90° elbow angle. Furthermore, the test was conducted by hanging statically at 90° in the elbow and shoulder joint. The angles were measured with a protractor, and the loop used in the daisy chain was noted for same position on post-test. During the isometric pull-up test, a half-crimp grip with passive thumb (i.e., only allowed to rest passively on the rung) was used. Before starting the test, the participants conducted a pull-up to achieve the 90° elbow angle and maintained the position for 3 seconds to minimize all sway of the body. The test was initiated when the participant was in this standardized position. On signal from test leader, the participants were instructed to generate as much force as possible (trying to complete the pull-up) and maintain the force output until a stop signal was given from the test leader after 3–4 seconds. In advance, the participants were told not to lose tension or “catch speed”, (i.e., lowering their body) before initiating the maximum contraction. Three attempts were conducted with a two-minute break between each attempt. The data was registered using MuscleLab software (v. 10.4, Ergotest Innovation A/S, Porsgrunn, Norway) and the best measurement for each parameter in one single execution was used in the statistical analysis. Reliability data from our lab have shown an intra-class correlation (ICC) of 0.90 and coefficient of variation (CV) of 12.0% for peak force output and ICC = 0.97 and CV = 8.9% for the average force output.

#### MVC on fingerboard

**Figure 2:**
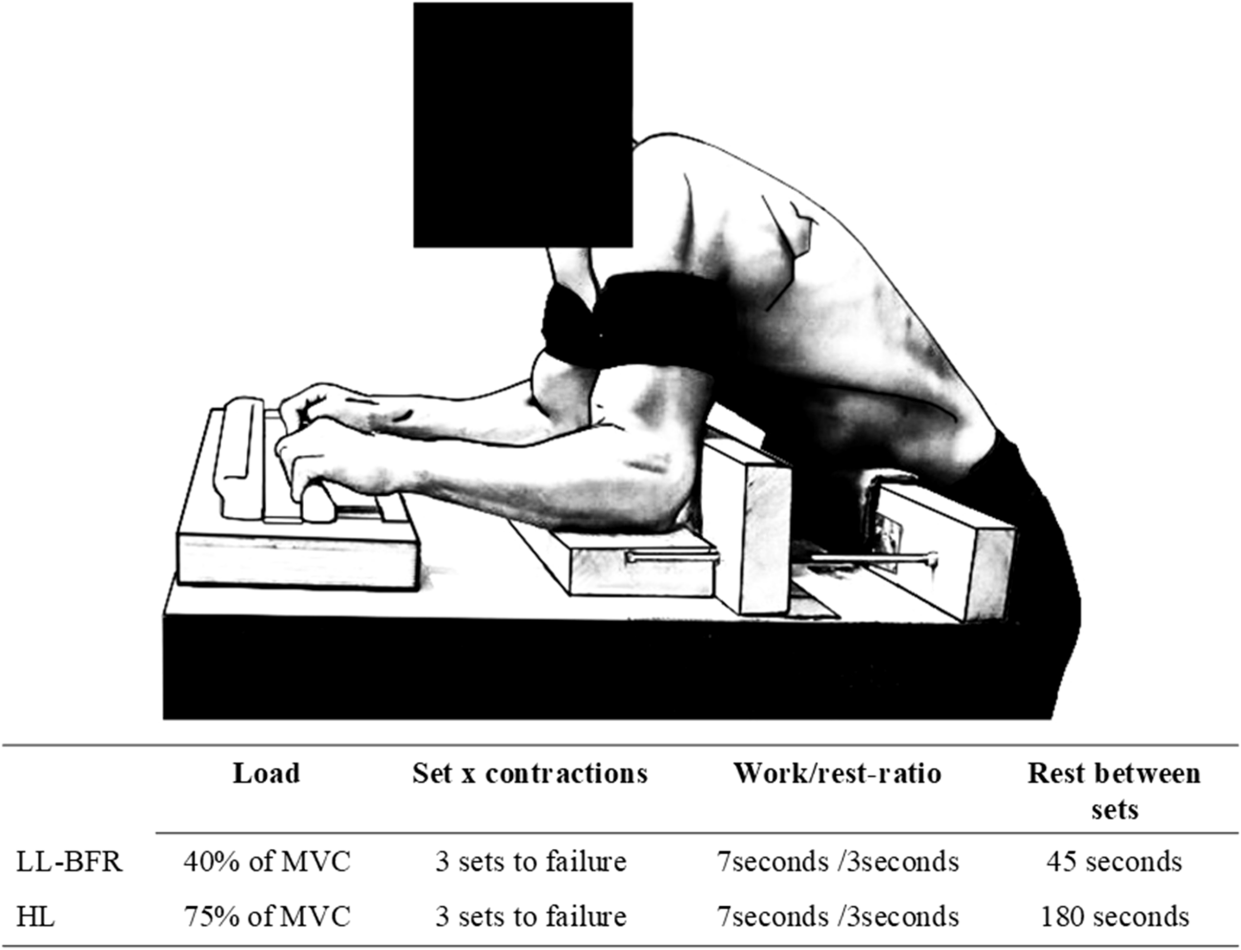
Overview of the two training interventions. LL-BFigure , low load blood flow restriction; HL, high load training; MVC, maximal voluntary contraction.

Five minutes after completing the isometric pull-up test the MVC test of the isolated finger flexors began. A custom-made apparatus was designed for MVC tests, and the same apparatus and set-up was used for the intervention training program. The set-up is shown in **Figure 2**, the participant in the figure have given his consent to publish the image in an online open access publication. The MVC was measured in the training device to assess the specific strength changes in the trained exercise. The apparatus consisted of a commercial fingerboard (Climbro, Sofia, Bulgaria) used in finger training- and testing consistent with a 23 mm rung and an embedded force sensor (70 Hz). The fingerboard was connected to a tablet with Bluetooth to monitor the generated force. The distance between the rung and elbows was adjusted to the individual arm length of the participants by moving the elbow lock with adjustable stacks to an elbow angle of 90-degree. The distance between the elbow lock and rung was adjusted to a point where the subject achieved a half-crimp grip on the rung, with angles of the distal and proximal interphalangeal joints of approximately 0-20˚ and 60-80˚ respectively. The participants were placed upright in front of the apparatus. The individual adjustments were noted and used at post-test and in training. The default “maximum strength test” setting in the app (Climbro) was used for the test. The MVC test consisted of two attempts with a one-minute rest between each attempt. The participants were instructed to conduct finger flexion as hard as possible for three seconds, without removing the elbows from the armrest. The highest result of the two attempts was recorded. The test was performed bilaterally, but if the subject pulled more than 113kg, the test was conducted unilaterally (due to the apparatus’ maximum load capacity). In these cases, the maximal loads of the left and right sides were combined for the analyses. Unpublished data from our lab has shown the MVC to have an ICC of 0.98 and CV = 3.1%.

#### Endurance test

The endurance test was conducted on the same apparatus and with the same individual adjustment as the MVC test. Furthermore, the test was performed as a bilateral intermittent test of one set to failure, with the workload on 57% of the most recently tested MVC. The workload (57%) was decided as it is the mean value between the two training loads (75% and 40% of MVC) in the intervention. This was chosen not to favor any of the training conditions. Each repetition consisted of seven seconds contraction followed by three seconds of rest (7:3 ratio) (Stien et al., 2019). During the isometric contractions, the force had to be within a ± five-percent deviation from the target force. The test was terminated when the force dropped below this target zone for two consecutive seconds. The performance was measured as total work, defined as kilograms multiplied by the number of seconds in the target zone. Data from our lab suggests the endurance test to have an ICC = 0.94 and a CV = 7.9%.

#### Climbing performance

Climbing performance was tested 15 minutes after the endurance test was completed. The test was conducted as lead climbing, on an indoor artificial climbing wall, in an eighteen-meter high and overhanging route consisting of 66 holds. The route was relatively uniform in character and did not contain any major cruxes. The route grade was French 6c (IRCRA 15). This grade was chosen for practical reasons, because a single route was used for all participants, and to ensure that each participant was able to complete minimal 50% of the route at the pre-test. If the participant topped out the route, the climber was lowered to the ground as fast as possible before the rope was dragged down. The participant then tied the rope to the harness and immediately continued climbing the route from the start. This process was repeated to the point of falling. The participants were allowed a maximum of six minutes per round to minimize the possibility of hanging on the best holds and resting for too long. The climbing performance was measured as the number of completed moves according to climbing competition rules. The participants were instructed to not (i.e., not allowed) climb the route between pre- and post-testing.

#### Training protocol

Both the LL-BFR and HL protocol were conducted in the test apparatus with identical individual adjustment as the MVC and endurance tests. Before each training session, the participant first conducted a 15–20-minute warm-up consisting of progressive easy to moderate bouldering. Secondly, the subject performed an MVC test (similar to the pre- and post-test) to adjust the target force for that specific training session. This was important to adjust for day-to-day variations and ensure a progression of the training load throughout the intervention period.

Both protocols consisted of three sets of repeated isometric contractions to failure. The intensity was set to 40% of MVC for LL-BFR and 75% of MVC for HL. Each repetition lasted seven seconds, followed by three seconds of rest. Each set was terminated when the force output dropped below the required intensity (± 5%) for two consecutive seconds. The rest periods between sets lasted 45 seconds for LL-BFR, and three minutes for HL, in line with recommendations and protocols in previous research (Lixandrão et al., 2018). When conducting the LL-BFR protocol, the participants were applied with inflatable cuffs (Occlude, Blood flow restriction exercise, Denmark, size s. (width: 9 cm x length: 38 cm)), placed as proximally as possible on the arms. The cuffs were inflated with a pressure equivalent to 60% of full arterial restriction (Loenneke et al., 2014; Patterson et al., 2019). The pressure was individualized for each subject prior to the first training bout using a Doppler apparatus (SonoTrax Series, Ultrasonic Pocket Doppler, Edan instruments Inc, China), as previously recommended (Patterson et al., 2019). The blood flow restriction was maintained throughout the entire training session including the rest periods. The participants were instructed not to lift their arms above heart level, to ensure minimum removal of venous blood from the blood restricted limbs. Finally, the pressure was controlled in each pause and adjusted if needed.

All participants were asked to register their training time outside the intervention and categorize it as either bouldering, lead climbing, strength training, or endurance training. To assist participants in categorizing their training, strength training was defined as traditional training using free weights, machines, body weight, or other equipment with the intention of improving muscular strength, while endurance training was defined as activities such as jogging, cycling, or swimming performed as either interval or continuous exercise. The average time (hours per week) per category was reported at pre- and post-test.

### Statistical analyses

All analyses were per-protocol and performed using SPSS (IBM Corp. Released 2020. IBM SPSS Statistics for Windows, Version 27.0. Armonk, NY: IBM Corp). A Shapiro-Wilk test of normality revealed that all variables (p = 0.087 – 0.686) except hypertrophy (p = 0.022) and performance (p = 0.001) were normally distributed. For the normally distributed data, between-groups differences were tested with an analysis of covariance (ANCOVA) using the results from pre-test as the covariate, whereas within-groups changes were addressed using a paired-samples t-test. Differences between the groups in training time outside the intervention were examined using an unpaired sample t-test. The non-parametric results were analyzed using a Wilcoxon signed rank test and a Mann–Whitney U test for the within- and between-groups comparisons, respectively. Statistical significance was accepted at p < 0.05 and the results are presented as means with standard deviations or median with interquartile range. Bonferroni corrections were applied to the within-group comparisons for peak force, average force, endurance, and MVC (treated as one family), by multiplying the p-values by four to mitigate Type I error inflation. Hedges’ g effect sizes (g) for the changes were calculated as the mean difference divided by the pooled and weighted standard deviations and interpreted as trivial (< 0.2), small (0.2 – 0.5), medium (0.5 – 0.8), and large (> 0.8) (Cohen, 1988). For the ANCOVA analyses, partial eta squared (η_p_^2^) was reported to indicate the proportion of variance explained by the intervention after adjusting for baseline values. Thresholds for interpreting η_p_^2^ were set at 0.01 (small), 0.06 (medium), and 0.14 (large) (Cohen, 1988). In this way, Hedges’ g provides a standardized effect size for direct group comparisons, while η_p_^2^ reflects how much variance is accounted for when baseline differences are statistically controlled. Effect sizes for the non-parametric results are presented as product movement r (r) and were calculated by dividing the Z-scores by the square number of observations and interpreted as trivial (< 0.1), small (0.1 – 0.3), medium (0.3 – 0.5), and large (> 0.5) (Cohen, 1988). To examine the correlation between the changes in the finger flexor strength tests and the climbing performance, the Pearson correlation coefficient was used. Correlation values <0.3, between 0.3 and 0.5, between 0.5 and 0.7 and >0.7 were defined as very weak, weak, moderate and strong respectively (Cohen, 1988).

## Results

Comparisons of the baseline results revealed that the LL-BFR group had a higher peak force (g = 0.986, p = 0.040) than the HL group. The remaining variables were not statistically different between the groups at pre-test (g = 0.213 – 0.932, p = 0.051 – 0.638).

The ANCOVA displayed no significant difference between the groups at post-test for peak force (F (Wernbom et al., 2008; Medernach et al., 2024) = 0.942, η_p_^2^ = 0.056, p = 0.346, see **Table 2**) or average force (F (Wernbom et al., 2008; Medernach et al., 2024) = 0.019, η_p_^2^ = 0.001, p = 0.891) in the isometric pull-up when adjusting for the pre-test values. Nor were MVC (F (Wernbom et al., 2008; Medernach et al., 2024) = 0.480, η_p_^2^ = 0.029, p = 0.498) or endurance (F (Wernbom et al., 2008; Medernach et al., 2024) = 0.413, η_p_^2^ = 0.025, p = 0.529) different between the groups at post-test.

**Figure 3:**
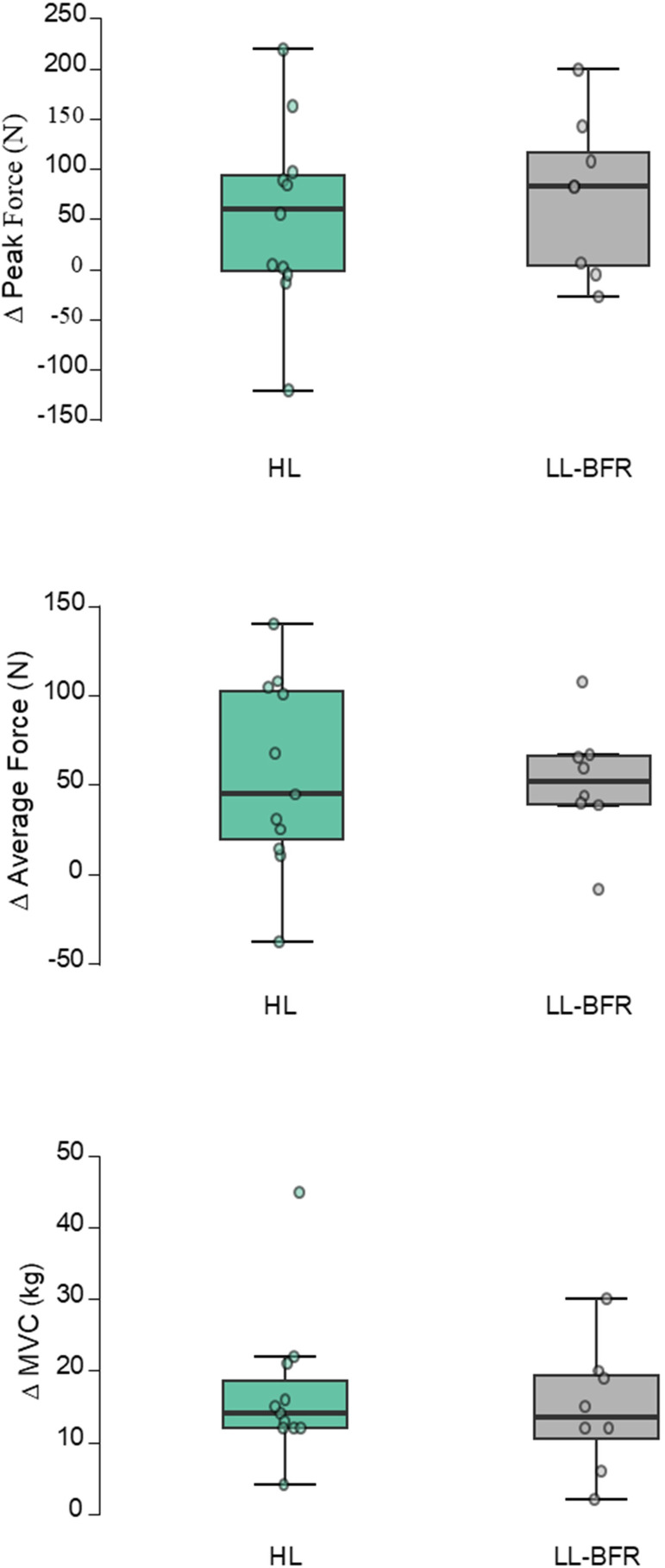
Changes from pre- to post-test; Individual (jitter), median (central horizontal line), interquartile range (box) and range (vertical lines) from pre- to post-test in the maximal strength tests (peak force, average force and maximal voluntary contraction (MVC)).LL-BFigure, low load blood flow restriction; HL, high load training; N, newton, kg, kilogram.

Within-groups comparisons revealed that the LL-BFR group improved average force (51.6 ± 32.9 N, g = 1.480, p = 0.012, see **Table 2**; **Figure 3**), but not peak force (73.4 ± 78.2 N, g = 0.888, p = 0.132) in the isometric pull-up. The same group improved MVC (14.5 ± 8.7 kg, g = 1.572, p = 0.008), but not endurance (g = 0.314, p = 1.000). The HL group improved average force (55.3 ± 53.5 N, g = 0.994, p = 0.024), but not peak force (g = 0.537, p = 0.376) in the isometric pull-up. The HL group also increased MVC (16.9 ± 10.5 kg, g = 1.552, p = 0.002), but not endurance (g = 0.410, p = 0.752).

**Table 2:** Pre-, post- and between groups effect size-values (at post-test) for both intervention groups.

Parameters	LL-BFR		HL		
	Pre	Post	Pre	Post	ES at post
Maximal strength					
Isometric pull-up					ηp^2^
Peak force (N)	366 ± 147	439 ± 188	490 ± 97	542 ± 148	0.056
Average force (N)	271± 152	323 ± 153*	362 ± 121	418 ± 151*	0.001
MVC (kg)	78.6 ± 24.0	93.1 ± 26.6#	90.3 ± 15.6	107.2 ± 14.5#	0.029
Endurance					
Total work (kg*s)	5560 ± 1778	6152 ± 1589	6012 ± 2181	6909 ± 2227	0.025
Hypertrophy					r
Circumference (cm)	26.6 ± 3.8	27.4 ± 4.0*	28.0 ± 1.9	28.6 ± 1.7*	0.379
Climbing performance					
Number of holds (n)	35.0 ± 61.5	48.5 ± 119.8*	49.0 ± 67.0	59.0 ± 88.0#	0.095

The normally distributed data (maximal strength and endurance) are presented as mean ± standard deviation and partial eta squared (ηp^2^) while the not normally distributed data (hypertrophy and climbing performance) are presented as median ± interquartile range and product movement r (r).

LL-BFigure, low load blood flow restriction; HL, high load training; N, newton; kg, kilogram; s, seconds; cm, centimeters; n, number, * significantly different from pre (p < 0.05), # significantly different from pre (p < 0.01).

**Figure 4:**
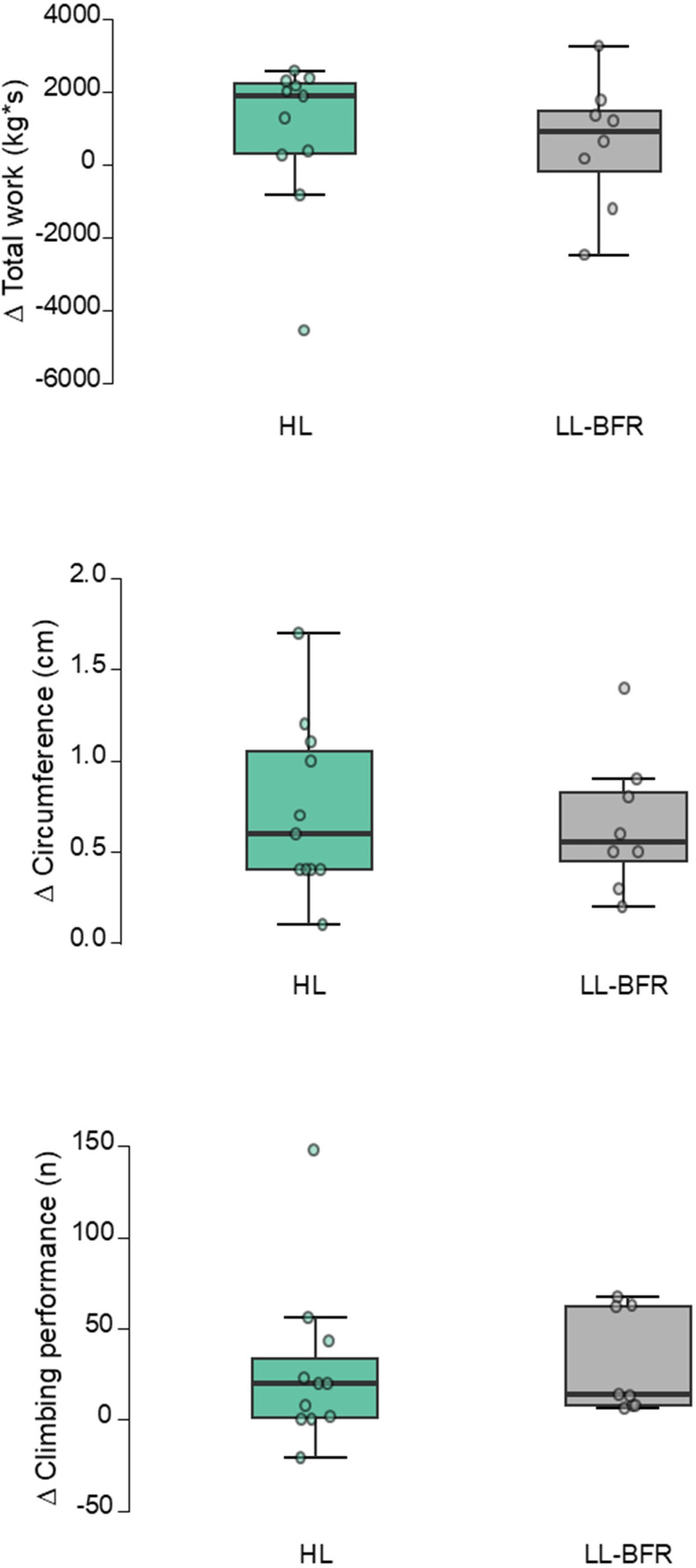
Changes from pre- to post-test; Individual (jitter), median (central horizontal line), interquartile range (box) and range (vertical lines) in the endurance-, hypertrophy- and climbing performance test.LL-BFigure, low load blood flow restriction; HL, high load training; s, seconds; kg, kilogram; cm, centimeter; n, number.

Regarding the non-parametric results, the LL-BFR group increased the hypertrophy (median) by 0.8 cm (r = 0.158, p = 0.012, see **Table 2**; **Figure 4**), and improved climbing performance by 13.5 moves from pre- to post-test (r = 0.210, p = 0.012). The HL group increased hypertrophy from pre- to post-test by 0.6 cm (r = 0.287, p = 0.038), and improved climbing performance by 10 moves (r = 0.105, p = 0.003). Neither hypertrophy (r = 0.379, p = 0.090) or performance (r = 0.095, p = 0.590) were different between the groups at post-test.

There were no significant correlations between the changes in the isometric pull-up tests and climbing performance (Pearson r = -0.005 – 0.177, p = 0.469 – 0.983), however a moderate correlation was observed between the changes in the MVC- test and climbing performance (Pearson r = 0.620, p = 0.005).

There were no significant differences between the groups regarding hours spent per week training outside the intervention (HL vs LL-BFR: Bouldering; 2.3 ± 1.8 vs 2.1 ± 1.2, g = 0.101, p = 0.822, Lead climbing; 3.1 ± 2.1 vs. 2.4 ± 2.1, g = 0.334, p = 0.462, Strength training; 1.1 ± 2.1 vs. 1.4 ± 1.3, g = 0.168, p = 0.710, Endurance training; 3.4 ± 2.6 vs. 3.5 ± 2.9, g = 0.017, p = 0.970). Furthermore, there were no significant differences within the groups from pre- to post-test for the same parameters (g = 0.056 – 0.581, p = 0.107 – 0.845).

## Discussion

The main finding of the present study was that both LL-BFR and HL interventions improved average force in the isometric pull-up, finger flexor MVC strength, circumference of the forearm and number of moves in the climbing performance test. Therefore, using LL-BFR appears as a potential alternative to HL training to improve maximal strength, forearm hypertrophy and climbing performance in advanced climbers.

The similar findings in maximal strength (MVC and average force output) and hypertrophy between the LL-BFR and HL resistance training are in line with the conclusion of a recent meta-analysis (Grønfeldt et al., 2020). Resistance exercise generates a number of physiological stimuli, including motor unit recruitment, endocrine responses, and satellite cell activation, and the sum of all stimuli appears to have been similar between high-load training and low-load BFR in our study (Hwang and Willoughby, 2019; Wernbom and Aagaard, 2020). Furthermore, both interventions had similar increases in hypertrophy (i.e., forearm circumference) which has been suggested to be strongly correlated to maximal strength (Stone et al., 2005). We also observed a strong relationship between hypertrophy and the different maximal strength measurements (r = 0.75 – 0.79, not included in the analyses).

Notably, both LL-BFR and HL improved strength in the MVC and average force output in the isometric pull-up, whereas peak force output did not improve. The effect sizes (g = 0.54 (HL) and 0.89 (LL-BFR)) imply that many of the participants had an improvement in the pull-up force, but might not reach statistical significance due to the error of the measurement. Based on the data from our lab, peak force output in the isometric pull-up is the measure with the lowest ICC (0.90) and highest CV (12%) among the three measurements. Hence, the measurement may not be sensitive enough to be able to detect true differences with the sample size included in the present study.

Both intervention groups improved their climbing performance. Although several factors influence climbing performance (Sanchez et al., 2019; Vasile et al., 2023; Medernach et al., 2024), previous research has identified finger flexor strength as one of the most important physical factors for climbing performance (Saul et al., 2019; Diez-Fernández et al., 2023). Furthermore, we observed a moderate correlation between the change in the MVC finger flexor test and the change in the climbing test. Our findings pinpoints the importance of focusing on finger flexor strength in the training schedule of advanced-level climbers and could most likely be explained by enhanced ability to control the body in ascending movement and maintain a firm grip to the different holds (Saul et al., 2019; Diez-Fernández et al., 2023). Of note, we did not observe similar correlations between climbing performance and the two measurements in the isometric pull-up test which may be explained by the greater variability (ICC and CV) in these tests compared. Finally, and importantly, these correlations involve only 19 participants and should therefore be interpreted with caution.

Three participants in the LL-BFR group withdrew from the study. They all reported discomfort during training as the main reason or one of the main reasons for the withdrawal. As all withdrawals occurred in the same group this introduces a risk of attrition bias, meaning that the dropouts may have influenced the study results. Given the relatively small sample size at pre-test, the drop-outs constituted a substantial proportion of the LL-BFR group (27%). Furthermore, the per-protocol statistical approach reflects only the participants who completed the intervention. This should be considered when interpreting our findings. This also highlights the importance of emphasizing adequate statistical power in future studies comparing LL-BFR and HL. Furthermore, a previous study from our lab comparing the perceptional responses between BFR and heavy resistance training of the finger flexors in advanced climbers reported that the BFR protocol was perceived as more discomforting with an effect size corresponding to a large effect (Rate of perceived discomfort scale: 9 vs 6, r = 0.66) (Andersen et al., 2023). This aligns with other studies examining other muscle groups that have also reported BFR to be perceived as more discomforting than traditional resistance training (Bell et al., 2018; Dankel et al., 2019). From a practical point of view, these findings emphasize the importance of considering the perception of the training before implementing it in a prolonged training schedule.

The present study has several limitations. Firstly, it was designed as a pilot study without performing a sample size calculation. Due to potential confounders such as biological variability, measurement error and regression towards the mean, the study may be too underpowered to detect differences between the groups. Consequently, future studies need an *a priori* sample size calculation. Furthermore, our participants were active climbers on an advanced level and the findings can not necessarily be generalized to other populations. Also, the sex-ratio was skewed with only two females included. However, analyzing without females yielded similar results. There was also some heterogeneity between the groups at pre-tests, although only reaching a statistically significant difference for peak force in the isometric pull-up test. Importantly, we analyzed the data using ANCOVA with the pre-test values as a covariate, which should account for these differences. Further, although we included a climbing test, we did not differentiate the route between the participants. This led to considerable between subject differences in terms of the number of moves climbed. Moreover, it could be argued that a bouldering test would have been more specific to the intervention, focusing on maximal strength. The reason for our choices was more due to practical considerations as it would be impossible for us to prepare a route for all the different climbers involved in the project and that the IRCRA-scale which was used in the inclusion criteria was based on lead climbing performance. However, we do acknowledge that testing climbing performance in both disciplines (i.e., lead and bouldering) and having several routes with different difficulties would have strengthened the design of the study. Also, future studies should differentiate the routes used in the performance test (for example by using Kilter boards) to match the individual level of the participants. Although we did control for training outside the intervention, this was limited to training volume (hours per week). Therefore, differences in factors such as load and intensity may have influenced the results. In addition, the participants did not perform any familiarization before the pre-tests. This may have introduced a learning effect, and led to overestimating the real effect of the intervention. Importantly, the aim of the study was to compare between-group differences, and this question should not be affected by the lack of familiarization. Finally, the intervention lasted only five weeks, which is rather short in terms of a training intervention. Especially for hypertrophy, five weeks of training may be too short to detect differences. Furthermore, hypertrophy was measured using circumference which is an indirect measure and should therefore be interpreted with caution. Importantly based on the reported perception of BFR (Andersen et al., 2023), we did not want to prolong the training in this pilot study more than necessary. Also, five weeks is within the range of a typical block in a periodized training regime (Issurin, 2016).

In summary, the results of the present study suggest that both low load BFR and high load resistance training are viable options for increasing the finger flexors strength and improving climbing performance. Low load BFR of the finger flexors could be a viable option in periods with high training volumes in terms of climbing, to reduce the mechanical loading of the finger flexors. This could potentially reduce the risk of overuse injuries. This could be of particular interest to coaches or active climbers at an advanced-level aiming to increase their performance. Importantly, one should be aware of the high perception of discomfort previously reported with BFR-training (Bell et al., 2018; Dankel et al., 2019; Andersen et al., 2023) as also reflected by the drop-out rate in the present study. Consequently, it might be most suitable for climbers at a certain level, particularly those that climb frequently, and are better able to tolerate the discomfort of BFR. In conclusion, within the limitations of this pilot study, no statistically significant differences in maximal strength, hypertrophy, endurance or climbing performance were observed between low load BFR and high load resistance training of the finger flexors in advanced level climbers. in. Future studies should ensure adequate statistical power to determine potential between group differences.

## Data Availability

The raw data supporting the conclusions of this article will be made available by the authors, without undue reservation.
